# Supplementary material for: Maturation of selected human mitochondrial tRNAs requires deadenylation
Source: eLife. 2017 Jul 26;6:e27596. doi: 10.7554/eLife.27596 (PMC5544427; doi:10.7554/eLife.27596)
Supplement: Supplementary file 2 . — DOI: http://dx.doi.org/10.7554/eLife.27596.018 [file elife-27596-supp2.docx]

**Supplementary File 2 | Antibodies and vectors**

| Antibody | Company |
| --- | --- |
| Rabbit polyclonal antibody to human PDE12 | Abcam (ab87738) |
| Mouse monoclonal Anti-FLAG M2 | Sigma-Aldrich (F3165) |
| Mouse polyclonal antibody cocktail to human OXPHOS components | Abcam (ab110411) |
| Rabbit polyclonal antibody to human mtPAP | Abcam (ab156684) |
| Rabbit polyclonal antibody to human bL12 | Proteintech Group (14795-1-AP) |
| Rabbit polyclonal antibody to human uL17 | Sigma-Aldrich (HPA043666) |
| Rabbit polyclonal antibody to human bL19 | Proteintech Group (16517-1-AP) |
| Rabbit polyclonal antibody to human uL23 | Proteintech Group (11706-1-AP) |
| Rabbit polyclonal antibody to human mL46 | Atlas Antibodies (HPA050116) |
| Rabbit polyclonal antibody to human uS17 | Proteintech Group (18881-1-AP) |
| Rabbit polyclonal antibody to human mS18b | Proteintech Group (16139-1-AP) |
| Rabbit polyclonal antibody to human mS35 | Proteintech Group (16457-1-AP) |
| Mouse monoclonal antibody to human beta-actin | Sigma-Aldrich (A2228) |
| Rabbit polyclonal antibody to human TOM20 | Santa Cruz Biotechnology (sc-11415) |
| Anti-rabbit IgG-HRP | Promega (W4011) |
| Anti-mouse IgG-HRP | Promega (W4021) |
| Alexa Fluor 594 Goat anti-Rabbit IgG | Thermo Fisher Scientific/ Molecular Probes (A-11012) |
| Alexa Fluor 488 Goat anti-Mouse IgG | Thermo Fisher Scientific/ Molecular Probes (A-11001) |

| Vectors | Company |
| --- | --- |
| pOG44 Flp-Recombinase Expression Vector | ThermoFisher Scientific (V600520) |
| pcDNA5/FRT/TO | ThermoFisher Scientific (V652020) |
| pZFN1/pZFN2 (CompoZr zinc finger nucleases) | Sigma-Aldrich |
