# Supplementary material for: Maturation of selected human mitochondrial tRNAs requires deadenylation
Source: eLife. 2017 Jul 26;6:e27596. doi: 10.7554/eLife.27596 (PMC5544427; doi:10.7554/eLife.27596)
Supplement: Supplementary file 3. — Sequences of DNA oligonucleotides for use in radioactive MPAT assay DOI: http://dx.doi.org/10.7554/eLife.27596.019 [file elife-27596-supp3.docx]

**Supplementary File 3 | Oligonucleotide sequences**

**Sequences of DNA oligonucleotides for use in radioactive MPAT assay**

| **Oligonucleotide name** | **Oligonucleotide sequence** |
| --- | --- |
| LIGN | [Phos]-ATG TGA GAT CAT GCA CAG TCA TA-[SpcC3] |
| ANTI-LIGN | GAC TGT GCA TGA TCT CAC AT |
| MPAT_12S_Fw1 | ACC TGG CGG TGC TTC ATA TC |
| MPAT_12S_Fw2 | AAG TGT ACT GGA AAG TGC AC |
| MPAT_16S_Fw1 | ACC CAA CCT CCG AGC AGT AC |
| MPAT_16S_Fw2 | TAT ACC CAC ACC CAC CCA AG |
| MPAT_ND1_Fw1 | CCG AAC TAG TCT CAG GCT TC |
| MPAT_ND1_Fw2 | TAC AAT CTC CAG CAT TCC CC |
| MPAT_ND2_Fw1 | CAC CCT TAA TTC CAT CCA CC |
| MPAT_ND2_Fw2 | CTA TCT CCC CTT TTA TAC |
| MPAT_ND3_Fw1 | TGC GGC TTC GAC CCT ATA TC |
| MPAT_ND3_Fw2 | GAC TAC AAA AAG GAT TAG AC |
| MPAT_ND4/4L_Fw1 | TG ACT TCT AGC AAG CCT CGC |
| MPAT_ND4/4L_Fw2 | ACC CCG ACA TCA TTA CCG G |
| MPAT_ND5_Fw1 | CAT GCC TCA GGA TAC TCC TC |
| MPAT_ND5_Fw2 | CAT CGT TGT ATT TCA ACT AC |
| MPAT_CYTB_Fw1 | TTC TCC GAT CCG TCC CTA AC |
| MPAT_CYTB_Fw2 | TAA TTG AAA ACA AAA TAC TC |
| MPAT_CO1_Fw1 | GAC CAA ACC TAC GCC AAA ATC |
| MPAT_CO1_Fw2 | CCA ACC CCA TGG CCT CCA TG |
| MPAT_CO2_Fw1 | ATT CCT AGA ACC AGG CGA CC |
| MPAT_CO2_Fw2 | TAT AGC ACC CCC TCT ACC CC |
| MPAT_CO3_Fw1 | GTC TCT ATT TTA CCC TCC TAC |
| MPAT_CO3_Fw2 | TGT ATG TCT CCA TCT ATT GAT G |
| MPAT_ATP86_Fw2 | CCC ACT TCT TAC CAC AAG GC |
| MPAT_ATP86_Fw2 | AGT AAG CCT CTA CCT GCA CG |
| MPAT_Lys_Fw1 | ACT GTA AAG CTA ACT TAG CAT TAA CC |
| MPAT_Lys_Fw2 | AAG AGA ACC AAC ACC TCT TTA C |
| MPAT_Glu_Fw1 | GTT CTT GTA GTT GAA ATA CAA CGA TG |
| MPAT_Glu_Fw2 | GAT GGT TTT TCA TAT CAT TGG TC |
| MPAT_Met_Fw1 | GTA AGG TCA GCT AAA TAA GCT ATC GG |
| MPAT_Met_Fw2 | TAT CGG GCC CAT ACC CCG AAA ATG |
| MPAT_SerAGY_Fw1 | GAG AAA GCT CAC AAG AAC TGC TAA C |
| MPAT_SerAGY_Fw2 | AAG AAC TGC TAA CTC ATG CC |
| MPAT_Val_Fw1 | CAG AGT GTA GCT TAA CAC AAA GCA CC |
| MPAT_Val_Fw2 | CAC CCA ACT TAC ACT TAG GAG |
| MPAT_His_Fw1 | TAA ATA TAG TTT AAC CAA AAC ATC AG |
| MPAT_His_Fw2 | ATC AGA TTG TGA ATC TGA CAA CAG |

**Sequences of DNA oligonucleotides for use in Next-generation sequencing MPAT assay for mitochondrial tRNAs**

| **Oligonucleotide name** | **Oligonucleotide sequence** |
| --- | --- |
| MPAT_LeuUUR_Fw | GTT AAG ATG GCA GAG CCC GGT AAT C |
| MPAT_Asn_Fw | TAG ATT GAA GCC AGT TGA TTA GGG TG |
| MPAT_Gln_Fw | TAG GAT GGG GTG TGA TAG GTG GCA C |
| MPAT_Phe_Fw | GTT TAT GTA GCT TAC CTC CTC AAA GC |
| MPAT_LeuCUN_Fw | AAG GAT AAC AGC TAT CCA TTG GTC |
| MPAT_Lys_Fw | ACT GTA AAG CTA ACT TAG CAT TAA CC |
| MPAT_His_Fw | TAA ATA TAG TTT AAC CAA AAC ATC AG |
| MPAT_Val_Fw | CAG AGT GTA GCT TAA CAC AAA GCA CC |
| MPAT_Ile_Fw | GAA ATA TGT CTG ATA AAA GAG TTA C |
| MPAT_Ala_Fw | AAG GGC TTA GCT TAA TTA AAG TGG C |
| MPAT_Glu_Fw | GTT CTT GTA GTT GAA ATA CAA CGA TG |
| MPAT_SerUCN_Fw | GAA AAA GTC ATG GAG GCC ATG GGG |
| MPAT_Pro_Fw | GAG AAT AGT TTA AAT TAG AAT CTT AG |
| MPAT_Met_Fw | GTA AGG TCA GCT AAA TAA GCT ATC GG |
| MPAT_Trp_Fw | GAA ATT TAG GTT AAA TAC AGA CCA AG |
| MPAT_Asp_Fw | GTA TTA GAA AAA CCA TTT CAT AAC TTT G |
| MPAT_Gly_Fw | CTT TTA GTA TAA ATA GTA CCG TTA AC |
| MPAT_Thr_Fw | GTC CTT GTA GTA TAA ACT AAT ACA CC |
| MPAT_Tyr_Fw | GGT AAA ATG GCT GAG TGA AGC ATT GG |
| MPAT_Cys_Fw | AGC TCC GAG GTG ATT TTC ATA TTG |
| MPAT_Arg_Fw | GGT ATA TAG TTT AAA CAA AAC GAA TG |
| MPAT_SerAGY_Fw | GAG AAA GCT CAC AAG AAC TGC TAA C |

**Primers and probe sequences for Q-PCR**

| **Oligonucleotide name** | **Oligonucleotide sequence** |
| --- | --- |
| B2M F | TGC TGT CTC CAT GTT TGA TGT ATC T |
| B2M R | TCT CTG CTC CCC ACC TCT AAG T |
| B2M probe | 6-Fam-TTG CTC CAC AGG TAG CTC TAG GAG G-Tamra |
| mt3211 F | CAC CCA AGA ACA GGG TTT GT |
| mt3298 R | TGG CCA TGG GTA TGT TGT TAA |
| mt3242 Probe | 6-Fam-TTA CCG GGC TCT GCC ATC T-Tamra |
| mt9827 F | CGT CAT TAT TGG CTC AAC |
| mt9974 R | GAT GGA GAC ATA CAG AAA TAG |
| mt9852 Probe | 6-Fam-ACT ATC TGC TTC ATC CGC CAC TAA-Tamra |

**DNA oligonucleotides used for construction of radiolabelled dsDNA probes**

| **Transcript** |  | **Oligonucleotide sequence** |
| --- | --- | --- |
| 12S | F | CAC TGA AAA TGT TTA GAC GGG |
|  | R | GGC TCC TCT AGA GGG ATA TG |
| 16S | F | TAG ATA TAG TAC CGC AAG GG |
|  | R | GAC TTG TTG GTT GAT TGT AG |
| ND1 | F | AAC CTC AAC CTA GGC CTC C |
|  | R | AAT GCT AGG GTG AGT GGT AGG |
| ND2 | F | TCC CAG AGG TTA CCC AAG |
|  | R | GAG TAG TGT GAT TGA GGT GGA G |
| ND4L/ND4 | F | ACT ACC ACT GAC ATG ACT TTC C |
|  | R | GGA GTC ATA AGT GGA GTC CG |
| ND5 | F | GTA GCA TTG TTC GTT ACA TGG |
|  | R | ACT GCT GCG AAC AGA GTG |
| CO1 | F | CTT ATT CGA GCC GAG CTG |
|  | R | GGT ATA GAA TGG GGT CTC CTC |
| CO2 | F | GCG CAA GTA GGT CTA CAA GAC GC |
|  | R | GCA TGA AAC TGT GGT TTG CTC C |
| ATP8/6 | F | CCC ATA CTC CTT ACA CTA TTC C |
|  | R | GTT AGC GGT TAG GCG TAC |
| CYTB | F | CTA CCT TCA CGC CAA TGG |
|  | R | TTT GTT AGG GAC GGA TCG |

**DNA oligonucleotides used for construction of template to produce radiolabelled ssRNA probes**

| **Transcript** |  | **Oligonucleotide sequence** |
| --- | --- | --- |
| ND3 | F | ATA AAC TTC GCC TTA ATT TTA ATA ATC |
|  | R | TAA TAC GAC TCA CTA TAG GGA TTC GGT TCA GTC TAA TCC TTT TTG TAG |
| ND6 | F | GGG GTT TTC TTC TAA GCC TTC |
|  | R | TAA TAC GAC TCA CTA TAG GGC CCC CGA GCA ATC TCA ATT AC |
| COX3 | F | CCT GAG AAC CAA AAT GAA CG |
|  | R | TAA TAC GAC TCA CTA TAG GGG CCA GGG CTA TTG GTT GAA TG |

**Oligonucleotides for production of MitoRibo-Seq libraries**

| **Oligonucleotide name** | **Oligonucleotide sequence** |
| --- | --- |
| Preadenylated 3’-adaptor | rATGGAATTCTCGGGTGCCAAGG |
| 5’ adaptor | GUUCAGAGUUCUACAGUCCGACGAUC |
| RT_Primer | GCCTTGGCACCCGAGAATTCCA |
| RP1 | AATGATACGGCGACCACCGAGATCTACA CGTTCAGAGTTCTACAGTCCGA |
| RPIX (Where N is primer number) | CAAGCAGAAGAC GGCATACGAGAT N6GTGACT GGAGTTCCTTGGCACCCGAGAATTCCA |
